# Targeting PCSK9 and Beyond for the Management of Low-Density Lipoprotein Cholesterol

**DOI:** 10.3390/jcm12155082

**Published:** 2023-08-02

**Authors:** Farzahna Mohamed, Brett Mansfield, Frederick J. Raal

**Affiliations:** Division of Endocrinology and Metabolism, Department of Internal Medicine, Faculty of Health Sciences, University of the Witwatersrand, Johannesburg 2193, South Africa; farzahna.mohamed@wits.ac.za (F.M.); brett.mansfield@wits.ac.za (B.M.)

**Keywords:** dyslipidemia, bempedoic acid, PCSK9 inhibitors, ANGPTL3 inhibitors

## Abstract

Reducing low-density lipoprotein cholesterol (LDL-C) levels is crucial to the prevention of atherosclerotic cardiovascular disease (ASCVD). However, many patients, especially those at very high ASCVD risk or with familial hypercholesterolemia (FH), do not achieve target LDL-C levels with statin monotherapy. The underutilization of novel lipid-lowering therapies (LLT) globally may be due to cost concerns or therapeutic inertia. Emerging approaches have the potential to lower LDL-C and reduce ASCVD risk further, in addition to offering alternatives for statin-intolerant patients. Shifting the treatment paradigm towards initial combination therapy and utilizing novel LLT strategies can complement existing treatments. This review discusses innovative approaches including combination therapies involving statins and agents like ezetimibe, bempedoic acid, cholesterol ester transfer protein (CETP) inhibitors as well as strategies targeting proprotein convertase subtilisin/kexin type 9 (PCSK9) and angiopoietin-like protein 3 (ANGPTL3) inhibition. Advances in nucleic acid-based therapies and gene editing are innovative approaches that will improve patient compliance and adherence. These strategies demonstrate significant LDL-C reductions and improved cardiovascular outcomes, offering potential for optimal LDL-C control and reduced ASCVD risk. By addressing the limitations of statin monotherapy, these approaches provide new management options for elevated LDL-C levels.

## 1. Introduction

Low-density lipoprotein cholesterol (LDL-C) is a well-established causal factor of atherosclerotic cardiovascular disease (ASCVD), which remains the leading global cause of mortality. Reducing LDL-C has been shown to lower cardiovascular events. In real-world clinical practice, the use of statin monotherapy is common, but despite the maximal dose, only a low percentage of patients reach currently recommended LDL-C target levels, especially those with established ASCVD or at high risk of ASCVD, including patients with familial hypercholesterolemia (FH). Recommended targets include an LDL-C of less than 55 mg/dL (<1.4 mmol/L) for patients with established ASCVD or very high risk and an LDL-C less than 70 mg/dL (<1.8 mmol/L) for high-risk patients [1,2]. The DA VINCI registry showed that statin-based monotherapy was used in approximately 84% of patients who received lipid-lowering therapy (LLT), resulting in only 33% of patients achieving the LDL-C target [3]. Moreover, the FHSC registry in patients with FH revealed that only 2.7% of the patients achieved LDL-C levels <70 mg/dL (1.8 mmol/L) with LLT. Both these registries show that combination therapies are underutilized, ranging from 10% combination therapy in patients without FH to approximately 21% in those with FH, with data from the SWEDEHEART registry, showing that 90% of the patients, including those at very high risk of cardiovascular events and those who experienced a recent myocardial infarction, achieved LDL-C goals through combination therapy [2,3]. Hence, combination LLT, along with lifestyle measures, should be started as soon as possible and ideally at the time of diagnosis in high-risk patients [1,2] There is also a need for additional LLT, such as proprotein convertase subtilisin/kexin type 9 (PCSK9) inhibitors and angiopoietin-like 3 (ANGPTL3) inhibitors to achieve lower LDL-C targets. Promising opportunities arise from the emerging strategies in cardiovascular disease (CVD) prevention, as they can complement or even replace existing therapies (Table 1). The focus has broadened from ‘high-intensity statin therapy’ to ‘high-intensity LLT’ for optimal LDL-C management [4]. This review discusses the currently available treatment regimens, highlights treatment gaps and challenges, and describes the potential of novel and emerging therapeutic approaches.

## 2. LDL-C Lowering, Statins and Beyond

The efficacy of traditional LLT in individuals with homozygous FH (HoFH) is influenced by the remaining LDL-receptor (LDLR) function, which varies with different treatments. Hence, it is essential to develop agents that can lower LDL-C levels independently of LDLR pathways (Table 2).

### 2.1. Statins

Statins have a tried and tested history, and for most patients, they will be the initial treatment of choice for achieving LDL-C reduction [19]. However, a significant proportion of patients will be poorly adherent for a variety of reasons. According to data from clinical trials, as many as 25% of patients will report statin-related muscle side effects [20]. However, many “muscle problems” remain poorly defined in studies, making this a difficult adverse event to truly quantify. It is very likely that statin-related muscle symptoms are overstated and that the true prevalence is much lower [21,22]. Nevertheless, views may linger among individuals who have been prescribed statins as a result of negative public perceptions. Poor adherence to LLT leads to adverse ASCVD outcomes [23]. Consequently, additional non-statin LDL-C lowering therapies are needed to meet the LDL-C targets required to reduce the risk of ASCVD. Furthermore, upfront combination LLT has now been advocated for all patients at very high and extremely high risk of ASCVD [24].

### 2.2. Ezetimibe

Ezetimibe is a cholesterol absorption inhibitor and reduces the transportation of dietary cholesterol via the Nieman-Pick C1-like 1 protein (NPC1L1) in the gastro-intestinal tract. The addition of ezetimibe to statin therapy has demonstrated LDL-C lowering of up to 24% [25]. Further evidence of the benefits of NPC1L1 inhibition comes from naturally occurring inactivating mutations in the *NPC1L1* gene, which have been shown to reduce both LDL-C and ASCVD risk [26]. Ezetimibe has a favorable side effect profile and is generally considered the agent of choice for intensification of lipid-lowering in combination with statin therapy [27].

### 2.3. Bempedoic Acid

Bempedoic acid is a prodrug that is activated in the liver by acyl-CoA synthetase 1 (ACSVL-1) [28]. The active form of the drug inhibits the mevalonate pathway of cholesterol synthesis by inhibiting the enzyme ATP citrate lyase (ACLY), which lies upstream of HMG-CoA reductase, the rate-limiting enzyme targeted by statins. ACLY is involved in the cleavage of citrate to form both oxaloacetate and acetyl-CoA, with acetyl-CoA being an important substrate in both lipid and fatty acid synthesis [28]. Much like statins, inhibition of the cholesterol biosynthesis pathway leads to upregulation of the LDLR and subsequent lowering of circulating LDL-C. The lipid lowering effect of bempedoic acid was evaluated in a pooled analysis of four randomized controlled trials evaluating the efficacy and safety in different populations over a 12-week period [29]. Trials such as CLEAR Harmony and CLEAR Wisdom enrolled participants with established ASCVD and/or heterozygous FH (HeFH), most of whom were already taking high-intensity statins [5,29]. The addition of bempedoic acid led to a 16% further reduction in LDL-C in treated patients compared to only a 1.8% reduction in the placebo group [29]. Patients intolerant of statins were enrolled in the CLEAR Serenity and CLEAR Tranquility trials and demonstrated a greater LDL-C lowering of 23% in those receiving bempedoic acid compared to a 1.5% reduction in the placebo group [6,7,29].

The cardiovascular outcomes trial, CLEAR Outcomes, evaluated the efficacy of bempedoic acid in statin-intolerant patients compared to placebo [8]. The between-group LDL-C reduction was 21.1% in favor of those receiving bempedoic acid, leading to a reduction in major adverse cardiovascular events [8]. Additionally, bempedoic acid may have anti-inflammatory properties. The CLEAR Outcomes study showed a significant reduction in highly sensitive C-reactive protein (hsCRP) [8]. It remains unclear whether the cardiovascular outcomes observed in the trial are solely a result of LDL-C lowering or if the anti-inflammatory component also played a role.

Combination LLT with bempedoic acid has shown promising results. The combination of bempedoic acid, ezetimibe and atorvastatin resulted in a 63.6% reduction in LDL-C after 6 weeks [30]. Combination therapy of bempedoic acid and ezetimibe was found to be complementary, leading to LDL-C reductions of up to 38.8% in the subset of patients not using statins, as observed in the CLEAR Tranquility and CLEAR Serenity trials [31]. This value surpasses that achieved by either drug used as monotherapy.

The enzyme ACSVL-1 is primarily expressed in the liver and kidneys and is absent in skeletal muscle [32]. As a result, activation of bempedoic acid does not occur in muscle, avoiding the muscle-related side effects occasionally encountered when taking statins. However, being at the intersection of multiple metabolic pathways, bempedoic acid is not without its own side effects. It carries the risk of increased uric acid and gout, as well as acute kidney injury [33]. Nevertheless, given its demonstrated efficacy, bempedoic acid should be considered as an alternative to statins in patients who are genuinely intolerant to statins.

### 2.4. Cholesterol Ester Transfer Protein (CETP) Inhibitors

Cholesterol ester transfer protein (CETP) is a glycoprotein involved in the transfer of cholesterol esters from high-density lipoprotein (HDL) to LDL and very low-density LDL (VLDL). Additionally, CETP is involved in the transfer of triglyceride (TG) from triglyceride-rich lipoproteins (TRLs) to HDL and LDL. Naturally occurring CETP deficiency as a result of heterozygous and homozygous *CETP* mutations has been identified as one of the major causes of higher HDL-C levels and the resultant lower risk of ASCVD in Japan [34]. Population-based studies have also shown an inverse relationship between HDL-C and ASCVD [35]. However, the causal nature of low HDL-C in ASCVD remains unclear, with conflicting results from various Mendelian randomization studies [36].

The results of randomized controlled trials of CETP inhibitors to increase serum HDL-C for the prevention of ASCVD or progression of atherosclerosis have been disappointing, leading to the discontinuation of most drugs in this class [37,38,39]. However, while initially developed to elevate HDL-C, the mechanism by which LDL-C is lowered is now known to be due to an increase in the fractional catabolic rate of LDL-C and ApoB clearance [40].

The positive outcomes observed in ASCVD events with anacetrapib, (OR 0.091 CI: 0.85–0.97), have been largely attributed to the drug’s LDL-C-lowering effect rather than its impact on raising HDL-C levels [41]. The heterogenous reduction in LDL-C observed with different CETP inhibitors is believed to be due to differential drug binding, with some showing potential to improve LDL-C-lowering efficacy through alterations in binding properties and sites [42]. The newest and most potent CETP inhibitor, obicetrapib, demonstrated significant reductions in LDL-C (45%), ApoB (29.8%), non-HDL-C (44%), and Lp(a) (56.5%) when added to high-intensity statin therapy in a phase 2 study [43]. Furthermore, obicetrapib led to an increase in HDL-C (165%) and ApoA1 (47.8%) levels [43].

Therefore, the future role of newer CETP inhibitors may lie in their LDL-C-lowering benefits rather than their initially proposed capacity to elevate HDL-C levels. Obicetrapib is currently being evaluated in a phase 3 randomized controlled trial to assess its efficacy in relation to cardiovascular outcomes. The outcome of this study will likely determine whether this class of drugs will be incorporated into clinical practice as an additional therapy to reduce LDL-C levels and ASCVD risk.

### 2.5. Strategies Targeting PCSK9 Inhibition

Recent developments in genetics have identified novel targets for LLT. The *PCSK9* gene is important in cholesterol homeostasis as it encodes for a proprotein convertase in the subtilase subfamily [44]. PCSK9 binds and delivers the LDLR to the lysosome for degradation; hence, increased production of PCSK9 is associated with a decrease in LDLR activity, resulting in elevated LDL-C levels and vice versa [4,44]. This led to the development of targeted therapies that inhibit PCSK9, including the monoclonal antibodies (mAbs), evolocumab and alirocumab, as well as gene silencing therapy. These strategies offer novel therapeutics for hypercholesterolemia, as statins upregulate the expression of both LDLR and PCSK9, which may explain the relatively small incremental reduction in LDL-C with each doubling of the statin dose [44].

#### 2.5.1. Monoclonal Anti-PCSK9 Antibodies

Alirocumab and evolocumab are fully human mAbs targeting PCSK9 in plasma and block its binding to the LDLR, resulting in more available receptors for the clearance of circulating LDL particles [44]. These drugs are administered subcutaneously on a bimonthly or monthly basis and have been found to be efficacious, safe, and well-tolerated in both adults and children, with the most common adverse events being nasopharyngitis, influenza, upper respiratory tract infection, and headache [45,46,47,48]. While PCSK9 inhibitors are indicated for primary prevention in patients at very high risk or secondary prevention in those who do not achieve LDL-C targets on maximally tolerated doses of statin and ezetimibe, their efficacy is reduced in patients with homozygous or compound heterozygous double LDLR variant carriers [1,2]. In these patients, the LDL-C-lowering effect is greater in those with partially functioning LDLR compared to those with negative or null–-null LDLR mutations, suggesting that for PCSK9 inhibitors to be effective in FH, at least one non-null allele coding a partially functioning LDLR must be present [45,49,50,51].

Clinical trials have shown that evolocumab significantly reduces LDL-C levels in both HoFH and HeFH patients [49,50,52]. In HeFH patients, evolocumab reduced LDL-C levels by 60% compared to placebo when administered every 2 weeks (140 mg) or monthly (420 mg) [49]. In HoFH patients, evolocumab was only half as effecitive, reducing LDL-C levels by 31% (65) [50]. Long-term safety and efficacy of evolocumab was observed in patients with HoFH and severe HeFH, whereby LDL-C was reduced by 21% and 55%, respectively [45]. Similarly, bimonthly alirocumab was effective at reducing LDL-C in patients with HeFH and HoFH, with reductions of up to 58% and 36%, respectively [53,54]. Data from the HEYMANS registry, a prospective registry of adults initiating evolocumab in routine clinical practice in 12 European countries, focused on evolocumab treatment over 30 months, showed a sustained 58% LDL-C reduction within 3 months, and high patient continuation (>90% at 12 and 30 months of follow up). Approximately 60% achieved a ≥50% reduction from baseline at each follow-up visit [55].

The use of PCSK9 mAbs has also been shown to lower the risk of myocardial infarction, ischemic stroke, and coronary revascularization in patients with hypercholesterolemia [56]. Alirocumab was also associated with a lower risk of all-cause death [56]. The FOURIER trial demonstrated that evolocumab reduced the risk of major adverse cardiovascular events compared to a placebo, and the ODYSSEY OUTCOMES trial showed similar results with alirocumab, with greater benefit seen in patients with higher baseline LDL-C levels [57,58]. Newer PCSK9 mAbs such as lerodalcibep, tafolecimab, and recaticimab are also under development. However, these drugs require administration every 2 to 4 weeks, making newer therapies with less frequent administration a favourable alternative.

#### 2.5.2. Small Interfering RNA Directed against PCSK9

Small interfering RNA (siRNA) is a naturally occurring double-stranded RNA molecule which can serve as an effective therapeutic modality. It works by binding to a RNA-induced silencing complex (RISC) in the cytoplasm, where the passenger RNA loads onto the RISC complex, acting as a template for RISC to recognize the target mRNA and undergo cleavage [59,60].

Inclisiran is a subcutaneously delivered siRNA targeting hepatic PCSK9 synthesis, resulting in a dose-dependent reduction in PCSK9 of up to 60% after a single dose and up to 69% after two doses [9]. Data from the ORION 1 trial, in patients at high risk for ASCVD, showed a reduction in LDL-C levels of up to 42% and 53% after a single dose and second dose, respectively at 6 months, with observations at one-year follow-up showing that the two-dose 300 mg regimen produced the highest proportion of responders and the greatest mean reduction in LDL-C [9,10]. An investigation of the long-term efficacy of the ORION-3 trial, a 4-year open-label extension study of the ORION-1 trial, showed a sustained reduction in LDL-C and PCSK9, with a mean LDL-C reduction of 44% [11].

Inclisiran has also been shown to be efficacious in FH patients, with ~40% reduction in LDL-C, highlighting that PCSK9 inhibition is mainly dependent on the up-regulation of normally functioning LDLR and overrides the minor role of clearance of LDL-C via the up-regulation of dysfunctional LDLR [12,13,14]. Furthermore, inclisiran has also been shown to significantly reduce composite major adverse cardiovascular events; however, larger cardiovascular outcome trials, such as ORION-4 and VICTORION-2 are being conducted to confirm these findings [15]. Pre-specified secondary analysis also suggested a significant reduction in LDL-C in primary prevention patients; however, larger studies are needed to confirm this [16]. The effect of inclisiran on LDL-C levels appeared consistent across a range of subgroups, including those with and without diabetes, metabolic syndrome, renal impairment, and across varying geographic regions, which included Europe and South Africa [17]. Safety and efficacy data of inclisiran, with a twice-yearly administration, could improve long-term adherence, making it a suitable alternative to traditional LLT and PCSK9 mAbs in patients who cannot achieve target LDL-C, particularly in patients with FH.

#### 2.5.3. Antisense Oligonucleotide Targeting PCSK9

Antisense oligonucleotide (ASO) therapies target RNA and act through RNA cleavage or blockage, resulting in gene silencing. The PCSK9-targeted ASO, AZD8233 (NCT04641299), inhibits hepatic PCSK9 synthesis and causes substantial reductions in LDL-C. Data from the ETESIAN trial, a phase 2b dose-ranging study, demonstrated a dose-dependent reduction in LDL-C, Lp(a), and ApoB of 79%, 45% and 67%, respectively. The broad lipid-modifying effect of AZD8233 on atherogenic lipoproteins may provide an opportunity to reduce cardiovascular risk in patients with ASCVD beyond currently available medications [61]. Unfortunately, further development of AZD8233 has been halted, and it will not advance into phase 3 development.

#### 2.5.4. Oral PCSK9 Inhibitors

Oral PCSK9 inhibitors are currently under development and may prove to be a safe and acceptable alternative to mAbs and siRNAs targeting PCSK9, but this will require compliance with daily oral administration.

##### Oral Peptide Derivative

MK-0616 (NCT05261126) is an oral, macrocyclic peptide that binds to PCSK9. Results from phase I clinical trials have shown >90% reduction in free PCSK9, and a reduction in LDL-C of 65% [62,63]. Recent Phase 2b data conducted in 381 adults with hypercholesterolemia (LDL-C ≥70 mg/dL and ≤160 mg/dL (≥1.8 and ≤4.1 mmol/L)) and clinical ASCVD, or an ASCVD risk equivalent, showed promising results [64]. Participants were randomly assigned to one of four doses of MK-0616 or a placebo and were monitored for adverse events. All doses of MK-0616 demonstrated statistically significant reductions in LDL-C compared to the placebo. The reductions were dose dependent with up to a 61% reduction from baseline. The treatment was well tolerated, and the proportion of participants experiencing adverse events was similar across all treatment groups [64].

##### Oral Antisense Oligonucleotide

A chemically modified ASO to PCSK9, ION449 (AZD6615) that can be administered orally is also being developed. The ASO is co-formulated with sodium caprate to improve oral bioavailability. Repeated oral daily dosing in dogs resulted in a bioavailability of 7% in the liver, and target engagement was confirmed in rats and monkeys. Daily dosing of 15 mg is predicted to reduce circulating PCSK9 by 80% at steady state, supporting continued development of the drug for treating dyslipidemia [65].

## 3. LDL-C Lowering beyond PCSK9 Inhibition

Continuation of a PCSK9 inhibitor is advocated if the reduction in LDL-C with PCSK9-directed therapy exceeds 15%. However, if there is a poor response to PCSK9-targeted treatment, as seen particularly often in patients with HoFH, this therapy should be stopped and other treatment options should be considered, including LDLR-independent therapies and/or lipoprotein apheresis [2].

### 3.1. Strategies Targeting ANGPTL3

Angiopoietin-like protein 3 (ANGPTL3) is a protein expressed in the liver that inhibits endothelial lipase and lipoprotein lipase [44,66,67]. Elevated ANGPTL3 levels are associated with increased LDL-C levels, which in turn are linked to a higher risk of ASCVD [68,69]. Inhibition of ANGPTL3 results in increased activity of these lipases and a reduction in atherogenic lipoproteins, including LDL-C, TRLs and HDL-C. The exact mechanism of LDL-C lowering with ANGPTL3 inhibition is uncertain but appears to be dependent on EL with apparent uptake via non-LDLR-mediated mechanisms [3,70,71]. Targeting ANGPTL3 with mAbs or siRNA therapeutics is a promising approach to lower high TG, TRLs, and elevated LDL-C levels.

#### 3.1.1. Monoclonal Anti-ANGPTL3 Antibodies

Evinacumab, a mAb targeting ANGPTL3, has been approved for the treatment of HoFH in patients aged 5 and above [2]. It effectively lowers LDL-C levels by approximately 50% in HoFH and HeFH when used alongside maximally tolerated background LLT [2,18,71,72]. The LDL-C reduction is not influenced by the specific genetic defect because the mechanism of action of evinacumab is independent of LDLR function [72,73]. Long-term follow-up studies have shown sustained LDL-C reduction in both adults and children [2,74]. Evinacumab has also been shown to significantly reduce TG and VLDL-C levels in patients with moderate or severe hypertriglyceridemia [75]. In patients with very high LDL-C levels who are already receiving intensive treatment, including PCSK9-targeting medications, the addition of evinacumab can further lower LDL-C by approximately 50%, thus proving to be an early intervention strategy to significantly mitigate the risk of cardiovascular events, bringing it closer to the risk levels observed in the general population [3]. Nevertheless, there are limitations to consider, such as the requirement for monthly intravenous dosing and the possibility of developing autoantibodies [18,76].

#### 3.1.2. Small Interfering RNA Directed against ANGPTL3

SiRNA therapy targets ANGPTL3 mRNA and inhibits its translation through the use of double-stranded RNA and the AGO2-RISC complex. Chemical modifications to the nucleotides increase potency and duration, allowing for less frequent subcutaneous injections [60].

ARO-ANG3 is a N-acetyl galactosamine (GalNAc)-conjugated siRNA therapy targeted against ANGPTL3 mRNA that has shown potent therapeutic inhibition of ANGPTL3 [60]. Studies in healthy subjects and patients with hyperlipidemia have demonstrated a dose-dependent reduction in ANGPTL3 of up to 93%, with the nadir occurring 2 weeks after the second dose [77] Preliminary results of the ARCHES-2 study, a dose-finding study of ARO-ANG3, showed promising outcomes in subjects with mixed dyslipidemia. Reductions in ANGPTL3 levels (15–71%), triglycerides (53–59%), LDL-C (22–32%), and non-HDL (34–45%) were observed. Further reductions in LDL-C were observed after the second dose at week 12. The study suggests that maximum reductions in ANGPTL3 and LDL-C can be achieved with an initial dose followed by subsequent doses at day 29, and then 3-monthly [78]. These findings highlight the potential of ARO-ANG3 as a treatment option for a wide range of dyslipidemia, including pure hypercholesterolemia as seen in HoFH. Recent results of the ongoing phase 2 study investigating the use of multiple doses in patients with HoFH showed a mean reduction in ANGPTL3 of 83% and LDL-C of 48%, supporting further investigation of ARO-ANG3 in HoFH [77,78,79].

Multi-dose studies of ARO-ANG3 in patients with hyperlipidemia, including those with HeFH, have also shown promising results. Significant reductions in LDL-C were observed in patients with and without HeFH; these reductions were of 35% and 28%, respectively [77]. Overall, ARO-ANG3 demonstrates a favorable safety profile and has the potential to effectively treat severe dyslipidemia by targeting ANGPTL3 and reducing atherogenic lipoproteins. Other siRNA therapies directed against ANGPTL3, such as Solibinisran (LY-3561774; Eli-Lilly [NCT04644809]), are also in early phase development.

#### 3.1.3. Antisense Oligonucleotide Targeting ANGPTL3

Vupanorsen, a GalNac-modified ASO, specifically targeted hepatic ANGPTL3 mRNA, leading to a decrease in both the production and secretion of ANGPTL3 [80]. However, the drug was discontinued after disappointing Phase 2b results in the TRANSLATE-TIMI 70 trial [80]. While vupanorsen demonstrated a significant reduction in TG and non-HDL-C, the reductions in ApoB and LDL-C were only modest, particularly at the highest doses [80]. Currently, there are no ongoing development efforts for ASO-based therapies targeting ANGPTL3.

### 3.2. Inhibiting Microsomal Triglyceride Transfer Protein (MTP) and apoB100 Synthesis

Targeting the inhibition of apoB100 and apoB48 is an effective approach to reduce atherogenic lipoproteins. Mipomersen, which is an ASO inhibitor of apoB, and lomitapide, an inhibitor of microsomal triglyceride transport protein activity, result in approximately a 25% and 50% reduction in LDL-C, respectively. Both drugs have the potential to increase hepatic fat content and elevate transaminase levels; however, their long-term safety in this regard is uncertain. Consequently, their utilization is restricted in cases of severe hypercholesterolemia [81].

## 4. Future Directions of LDL-C Lowering

Therapies to lower LDL-C have progressed from the inhibition of proteins to the modification and inhibition of gene expression and the translation of their products. The focus has more recently shifted to the potential of gene-editing therapies.

### 4.1. Gene Editing—CRISPR

Targeting mRNA expression using siRNA and ASO therapies can temporarily inhibit the expression of the targeted protein. However, the emergence of genome-editing therapies offers the potential for once-off treatment in individuals at high and very high risk of ASCVD [82]. Various genome-editing strategies, including nuclease editing, base editing, and epigenome editing, have been developed for this purpose [82]. Among these strategies, the CRISPR/Cas system, discovered by Emmanuelle Charpentier and Jennifer Doudna in 2012, has become one of the most widely used genome-editing tools [83]. The CRISPR/Cas system utilizes a guide RNA and a Cas protein to introduce double-stranded breaks in the DNA [82]. Base editors, another type of genome-editing tool, also employ a guide RNA, but instead of introducing double-stranded breaks, they induce single-nucleotide changes that can disrupt gene structure and function [82,84]. The most commonly used base editors involve the replacement of cytosine with thymidine or the replacement of adenine with guanine [82]. These methods of genome editing, such as base editing and prime editing, are less likely to result in significant off-target effects, as they involve altering a single nucleotide [82].

The PCSK9 gene is considered a potential target for gene-editing therapy due to naturally occurring loss-of-function mutations that lead to lifelong low circulating LDL-C levels and an 88% relative risk reduction for ASCVD without adverse long-term effects [85]. PCSK9 is primarily expressed in the liver, making it an attractive candidate for editing [85]. Editing of the PCSK9 gene in primate models has shown promising results, with reductions of up to 90% in circulating PCSK9 and up to 60% in LDL-C levels [86,87,88]. However, concerns about off-target DNA cleavage have been raised, although no clinical consequences have been observed in a follow-up period of 3 years [89]. A phase 1 clinical trial is currently underway, evaluating the safety and efficacy of VERVE-101 (NCT05398029), a base editor targeting PCSK9, in patients with HeFH and established ASCVD.

The ANGPTL3 gene is another potential target for gene-editing therapies, as naturally occurring loss-of-function mutations in ANGPTL3 have no long-term health consequences and are associated with hypolipidemia and lower risk of ASCVD [90]. In a mouse model with HoFH, base editing of ANGPTL3 led to significant reductions in TC by 51% and TG by 56% [91]. However, no non-human primate studies have been reported thus far. While gene-editing therapies hold promise for durable lipid lowering, they will remain in the drug development pipeline until safety concerns regarding potential off-target effects are adequately addressed.

### 4.2. Vaccination

Vaccination has played a crucial role in reducing the incidence and prevalence of communicable diseases, but significant progress has also been made in developing vaccines for non-communicable chronic diseases like cancer, hypertension, and Alzheimer’s disease [92]. The objective of vaccination is to stimulate an adequate antibody response to neutralize specific proteins while minimizing T-cell activation to avoid unwanted tissue damage [93,94,95].

In murine models of HeFH, an anti-PCSK9 peptide vaccine resulted in a sustained 50% reduction in LDL-C levels over a one-year period [96]. Another approach involved vaccinating against PCSK9 using a virus-like particle (VLP) vaccine, which achieved a 10–15% decrease in LDL-C in rhesus macaques [97]. VLPs carry self-antigens on their surface, effectively overcoming B-cell tolerance and inducing a neutralizing antibody response [95]. Vaccination against ANGPTL3 in dyslipidemic obese mouse models (ob/ob mice) and FH mouse models demonstrated significant reductions in TG, VLDL-C, HDL-C, and LDL-C, with diminishing effects observed after 30 weeks [98,99].

Although mAbs targeting proteins like PCSK9 and ANGPTL3 have become part of the lipid-lowering armamentarium, vaccination to induce a sufficient host antibody response against specific proteins is preferred to relying on foreign-derived mAbs which require repeated administration [99]. Compared to passive immunotherapy with mAbs, vaccination offers advantages in terms of cost and dosing frequency. Mab therapy can be expensive and requires dosing every two to four weeks, while vaccination is a more cost-effective option, especially in resource-limited countries [99].

## 5. Conclusions

The effective treatment of LDL-C is crucial for reducing ASCVD and mortality, but many patients fail to achieve treatment goals. Knowledge of newer agents and their use is essential to reach treatment targets. Ongoing research, cardiovascular outcome data, and guideline recommendations are needed to improve ASCVD risk reduction. Advancements in genetic studies have identified new treatment targets, while the development of targeted therapies allows for personalized care and improved medication adherence.

## Figures and Tables

**Table 1:** Reducing ASCVD risk by lowering LDL-cholesterol.

Therapeutic Strategy	Therapies with Predominantly LDL-C Reduction and Supporting CVOT Trials		Therapies with LDL-C Plus TRL (Remnants) Reduction
Small molecules	Statins	CTCC *	
	Ezetemibe	IMPROVE-IT *	
	Bempedoic acid	CLEAR OUTCOMES *	
Monoclonal antibodies	**PCSK9-Inhibitors**	FOURIER *	**ANGPTL3-inhibitor**
		ODYSSEY OUTCOMES *	Evinacumab
Antisense oligonucleotides			**ANGPTL3-inhibitor**
			Vupanorsen
Small interfering RNA	**PCSK9-Inhibitors** Inclisiran	ORION-4 **	**ANGPTL3-inhibitor**
		VICTORION PREVENT-2 **	ARO-ANG3 Solbinsiran

* Completed Cardiovascular Outcome Trials (CVOT). ** Ongoing CVOT trials. A summary of current and emerging lipid lowering therapeutic strategies that reduce ASCVD risk by lowering LDL-C. ASCVD—atherosclerotic cardiovascular disease; LDL-C—low-density lipoprotein cholesterol; CVOT—cardiovascular outcome trial; TRL—triglyceride rich lipoproteins; CTCC—Cholesterol Treatment Trialists Collaboration; PCSK9—proprotein convertase subtilisin/kexin type 9; ANGPTL3—angiopoietin-like protein 3; RNA—ribonucleic acid.

**Table 2:** Impact of lipid lowering strategies in reducing low-density lipoprotein cholesterol.

Drug	Target	Efficacy of LDL-C Lowering
LDLR dependent		
**Statin**	HMG-CoA-reductase	~30–50% [4]
**Ezetimibe**	NPC1L1	~15–20% [4]
**Bempedoic acid**	ACLY	~17–25% [5,6,7,8]
**PCSK9-inhibitors** (Mab)	Circulating PCSK9	~50–60% [4]
**PCSK9-inhibitors** (siRNA)	Hepatic PCSK9 synthesis	~50% [9,10,11,12,13,14,15,16,17]
**LDLR independent**		
**Evinacumab**	Circulating ANGPTL3	~40–60% [18]

A summary of the target and effect of lipid-lowering therapies in decreasing LDL-C. LDL-C, low-density lipoprotein cholesterol; HMG-CoA-reductase, 3-hydroxy-3-methylglutaryl-coenzyme A; NPC1L1, Niemann-Pick C1 like 1; ACL, ATP citrate lyase; PCSK9, proprotein convertase subtilisin/kexin type 9; ANGPTL3, angiopoietin-like 3.

## Data Availability

Not applicable.

## References

[1] Visseren F.L.J., Mach F., Smulders Y.M., Carballo D., Koskinas K.C., Bäck M., Benetos A., Biffi A., Boavida J.M., Capodanno D. (2021). 2021 ESC Guidelines on cardiovascular disease prevention in clinical practice. Eur. Heart J..

[2] Cuchel M., Raal F.J., A Hegele R., Al-Rasadi K., Arca M., Averna M., Bruckert E., Freiberger T., Gaudet D., Harada-Shiba M. (2023). 2023 Update on European Atherosclerosis Society Consensus Statement on Homozygous Familial Hypercholesterolaemia: New treatments and clinical guidance. Eur. Heart J..

[3] Brandts J., Ray K.K. (2023). Novel and future lipid-modulating therapies for the prevention of cardiovascular disease. Nat. Rev. Cardiol..

[4] Tokgözoğlu L., Libby P. (2022). The dawn of a new era of targeted lipid-lowering therapies. Eur. Heart J..

[5] Ray K.K., Bays H.E., Catapano A.L., Lalwani N.D., Bloedon L.T., Sterling L.R., Robinson P.L., Ballantyne C.M. (2019). Safety and Efficacy of Bempedoic Acid to Reduce LDL Cholesterol. N. Engl. J. Med..

[6] Ballantyne C.M., Banach M., Mancini G.J., Lepor N.E., Hanselman J.C., Zhao X., Leiter L.A. (2018). Efficacy and safety of bempedoic acid added to ezetimibe in statin-intolerant patients with hypercholesterolemia: A randomized, placebo-controlled study. Atherosclerosis.

[7] Laufs U., Banach M., Mancini G.B.J., Gaudet D., Bloedon L.T., Sterling L.R., Kelly S., Stroes E.S.G. (2019). Efficacy and Safety of Bempedoic Acid in Patients with Hypercholesterolemia and Statin Intolerance. J. Am. Heart Assoc..

[8] Nissen S.E., Lincoff A.M., Brennan D., Ray K.K., Mason D., Kastelein J.J.P., Thompson P.D., Libby P., Cho L., Plutzky J. (2023). Bempedoic Acid and Cardiovascular Outcomes in Statin-Intolerant Patients. N. Engl. J. Med..

[9] Ray K.K., Landmesser U., Leiter L.A., Kallend D., Dufour R., Karakas M., Hall T., Troquay R.P., Turner T., Visseren F.L. (2017). Inclisiran in Patients at High Cardiovascular Risk with Elevated LDL Cholesterol. N. Engl. J. Med..

[10] Ray K.K., Stoekenbroek R.M., Kallend D., Nishikido T., Leiter L.A., Landmesser U., Wright R.S., Wijngaard P.L.J., Kastelein J.J.P. (2019). Effect of 1 or 2 Doses of Inclisiran on Low-Density Lipoprotein Cholesterol Levels: One-Year Follow-up of the ORION-1 Randomized Clinical Trial. JAMA Cardiol..

[11] Ray K.K., Troquay R.P.T., Visseren F.L.J., A Leiter L., Wright R.S., Vikarunnessa S., Talloczy Z., Zang X., Maheux P., Lesogor A. (2023). Long-term efficacy and safety of inclisiran in patients with high cardiovascular risk and elevated LDL cholesterol (ORION-3): Results from the 4-year open-label extension of the ORION-1 trial. Lancet Diabetes Endocrinol..

[12] Raal F.J., Kallend D., Ray K.K., Turner T., Koenig W., Wright R.S., Wijngaard P.L., Curcio D., Jaros M.J., Leiter L.A. (2020). Inclisiran for the Treatment of Heterozygous Familial Hypercholesterolemia. N. Engl. J. Med..

[13] Raal F.J., Sjouke B., Hovingh G.K., Isaac B.F. (2016). Phenotype diversity among patients with homozygous familial hypercholesterolemia: A cohort study. Atherosclerosis.

[14] Novartis Pharmaceuticals (2022). A Two-Part (Double-Blind Placebo Controlled/Open-Label) Multicenter Study to Evaluate Safety, Tolerability, and Efficacy of Inclisiran in Subjects with Homozygous Familial Hypercholesterolemia (Hofh) (ORION-5).

[15] Ray K.K., Raal F.J., Kallend D.G., Jaros M.J., Koenig W., A Leiter L.A., Landmesser U., Schwartz G.G., Lawrence D., Friedman A. (2022). Inclisiran and cardiovascular events: A patient-level analysis of phase III trials. Eur. Heart J..

[16] Ray K.K., Kallend D., A Leiter L., Raal F.J., Koenig W., Jaros M.J., Schwartz G.G., Landmesser U., Conde L.G., Wright R.S. (2022). Effect of inclisiran on lipids in primary prevention: The ORION-11 trial. Eur. Heart J..

[17] Ray K.K., Wright R.S., Kallend D., Koenig W., Leiter L.A., Raal F.J., Bisch J.A., Richardson T., Jaros M., Wijngaard P.L. (2020). Two Phase 3 Trials of Inclisiran in Patients with Elevated LDL Cholesterol. N. Engl. J. Med..

[18] Rosenson R.S., Burgess L.J., Ebenbichler C.F., Baum S.J., Stroes E.S., Ali S., Khilla N., Hamlin R., Pordy R., Dong Y. (2020). Evinacumab in Patients with Refractory Hypercholesterolemia. N. Engl. J. Med..

[19] Collins R., Reith C., Emberson J., Armitage J., Baigent C., Blackwell L., Blumenthal R., Danesh J., Smith G.D., DeMets D. (2016). Interpretation of the evidence for the efficacy and safety of statin therapy. Lancet.

[20] Ganga H.V., Slim H.B., Thompson P.D. (2014). A systematic review of statin-induced muscle problems in clinical trials. Am. Heart J..

[21] Reith C., Baigent C., Blackwell L., Emberson J., Spata E., Davies K., Halls H., Holland L., Wilson K., Armitage J. (2022). Effect of statin therapy on muscle symptoms: An individual participant data meta-analysis of large-scale, randomised, double-blind trials. Lancet.

[22] Wood F.A., Howard J.P., Finegold J.A., Nowbar A.N., Thompson D.M., Arnold A.D., Rajkumar C.A., Connolly S., Cegla J., Stride C. (2020). N-of-1 Trial of a Statin, Placebo, or No Treatment to Assess Side Effects. N. Engl. J. Med..

[23] Nielsen S.F., Nordestgaard B.G. (2016). Negative statin-related news stories decrease statin persistence and increase myocardial infarction and cardiovascular mortality: A nationwide prospective cohort study. Eur. Heart J..

[24] Ray K.K., Reeskamp L.F., Laufs U., Banach M., Mach F., Tokgözoğlu L.S., Connolly D.L., Gerrits A.J., Stroes E.S.G., Masana L. (2022). Combination lipid-lowering therapy as first-line strategy in very high-risk patients. Eur. Heart J..

[25] Cannon C.P., Blazing M.A., Giugliano R.P., McCagg A., White J.A., Théroux P., Darius H., Lewis B.S., Ophuis T.O., Jukema J.W. (2015). Ezetimibe Added to Statin Therapy after Acute Coronary Syndromes. N. Engl. J. Med..

[26] The Myocardial Infarction Genetics Consortium Investigators (2014). Inactivating Mutations in *NPC1L1* and Protection from Coronary Heart Disease. N. Engl. J. Med..

[27] Mach F., Baigent C., Catapano A.L., Koskinas K.C., Casula M., Badimon L., Chapman M.J., De Backer G.G., Delgado V., Ference B.A. (2020). 2019 ESC/EAS Guidelines for the management of dyslipidaemias: Lipid modification to reduce cardiovascular risk. Eur. Heart J..

[28] Pinkosky S.L., Newton R.S., Day E.A., Ford R.J., Lhotak S., Austin R.C., Birch C.M., Smith B.K., Filippov S., Groot P.H. (2016). Liver-specific ATP-citrate lyase inhibition by bempedoic acid decreases LDL-C and attenuates atherosclerosis. Nat. Commun..

[29] Banach M., Duell P.B., Gotto A.M., Laufs U., Leiter L.A., Mancini G.B.J., Ray K.K., Flaim J., Ye Z., Catapano A.L. (2020). Association of Bempedoic Acid Administration with Atherogenic Lipid Levels in Phase 3 Randomized Clinical Trials of Patients with Hypercholesterolemia. JAMA Cardiol..

[30] Rubino J., MacDougall D.E., Sterling L.R., Hanselman J.C., Nicholls S.J. (2021). Combination of bempedoic acid, ezetimibe, and atorvastatin in patients with hypercholesterolemia: A randomized clinical trial. Atherosclerosis.

[31] Laufs U., Ballantyne C.M., Banach M., Bays H., Catapano A.L., Duell P.B., Goldberg A.C., Gotto A.M., Leiter L.A., Ray K.K. (2022). Efficacy and safety of bempedoic acid in patients not receiving statins in phase 3 clinical trials. J. Clin. Lipidol..

[32] Ruscica M., Sirtori C.R., Carugo S., Banach M., Corsini A. (2022). Bempedoic Acid: For Whom and When. Curr. Atheroscler. Rep..

[33] Bays H.E., Banach M., Catapano A.L., Duell P.B., Gotto A.M., Laufs U., Leiter L.A., Mancini G.B.J., Ray K.K., Bloedon L.T. (2020). Bempedoic acid safety analysis: Pooled data from four phase 3 clinical trials. J. Clin. Lipidol..

[34] Inazu A., Jiang X.C., Haraki T., Yagi K., Kamon N., Koizumi J., Mabuchi H., Takeda R., Takata K., Moriyama Y. (1994). Genetic cholesteryl ester transfer protein deficiency caused by two prevalent mutations as a major determinant of increased levels of high density lipoprotein cholesterol. J. Clin. Investig..

[35] Danesh J. (2009). Major Lipids, Apolipoproteins, and Risk of Vascular Disease. JAMA.

[36] White J., Swerdlow D.I., Preiss D., Fairhurst-Hunter Z., Keating B.J., Asselbergs F.W., Sattar N., Humphries S.E., Hingorani A.D., Holmes M.V. (2016). Association of Lipid Fractions with Risks for Coronary Artery Disease and Diabetes. JAMA Cardiol..

[37] Kastelein J.J.P., van Leuven S.I., Burgess L., Evans G.W., Kuivenhoven J.A., Barter P.J., Revkin J.H., Grobbee D.E., Riley W.A., Shear C.L. (2007). Effect of Torcetrapib on Carotid Atherosclerosis in Familial Hypercholesterolemia. N. Engl. J. Med..

[38] Nissen S.E., Nicholls S.J., Shear C.L., Bachinsky W.B. (2007). Effect of Torcetrapib on the Progression of Coronary Atherosclerosis. N. Engl. J. Med..

[39] Schwartz G.G., Olsson A.G., Abt M., Ballantyne C.M., Barter P.J., Brumm J., Chaitman B.R., Holme I.M., Kallend D., Leiter L.A. (2012). Effects of dalcetrapib in patients with a recent acute coronary syndrome. N. Engl. J. Med..

[40] Nurmohamed N.S., Ditmarsch M., Kastelein J.J.P. (2022). Cholesteryl ester transfer protein inhibitors: From high-density lipoprotein cholesterol to low-density lipoprotein cholesterol lowering agents?. Cardiovasc. Res..

[41] The HPS3/TIMI55–REVEAL Collaborative Group (2017). Effects of Anacetrapib in Patients with Atherosclerotic Vascular Disease. N. Engl. J. Med..

[42] Schmidt A.F., Hunt N.B., Gordillo-Marañón M., Charoen P., Drenos F., Kivimaki M., Lawlor D.A., Giambartolomei C., Papacosta O., Chaturvedi N. (2021). Cholesteryl ester transfer protein (CETP) as a drug target for cardiovascular disease. Nat. Commun..

[43] Nicholls S.J., Ditmarsch M., Kastelein J.J., Rigby S.P., Kling D., Curcio D.L., Alp N.J., Davidson M.H. (2022). Lipid lowering effects of the CETP inhibitor obicetrapib in combination with high-intensity statins: A randomized phase 2 trial. Nat. Med..

[44] Ito M.K., Santos R.D. (2017). PCSK9 Inhibition with Monoclonal Antibodies: Modern Management of Hypercholesterolemia: PSCK9 in Management of Hypercholesterolemia. J. Clin. Pharmacol..

[45] Santos R.D., Stein E.A., Hovingh G.K., Blom D.J., Soran H., Watts G.F., López J.A.G., Bray S., Kurtz C.E., Hamer A.W. (2020). Long-Term Evolocumab in Patients with Familial Hypercholesterolemia. J. Am. Coll. Cardiol..

[46] Santos R.D., Ruzza A., Hovingh G.K., Wiegman A., Mach F., Kurtz C.E., Hamer A., Bridges I., Bartuli A., Bergeron J. (2020). Evolocumab in Pediatric Heterozygous Familial Hypercholesterolemia. New Engl. J. Med..

[47] Daniels S., Caprio S., Chaudhari U., Manvelian G., Baccara-Dinet M.T., Brunet A., Scemama M., Loizeau V., Bruckert E. (2020). PCSK9 inhibition with alirocumab in pediatric patients with heterozygous familial hypercholesterolemia: The ODYSSEY KIDS study. J. Clin. Lipidol..

[48] Santos R.D., Ruzza A., Hovingh G.K., Stefanutti C., Mach F., Descamps O.S., Bergeron J., Wang B., Bartuli A., Buonuomo P.S. (2022). Paediatric patients with heterozygous familial hypercholesterolaemia treated with evolocumab for 80 weeks (HAUSER-OLE): A single-arm, multicentre, open-label extension of HAUSER-RCT. Lancet Diabetes Endocrinol..

[49] Raal F.J., A Stein E., Dufour R., Turner T., Civeira F., Burgess L., Langslet G., Scott R., Olsson A.G., Sullivan D. (2015). PCSK9 inhibition with evolocumab (AMG 145) in heterozygous familial hypercholesterolaemia (RUTHERFORD-2): A randomised, double-blind, placebo-controlled trial. Lancet.

[50] Raal F.J., Honarpour N., Blom D.J., Hovingh G.K., Xu F., Scott R., Wasserman S.M., A Stein E. (2015). Inhibition of PCSK9 with evolocumab in homozygous familial hypercholesterolaemia (TESLA Part B): A randomised, double-blind, placebo-controlled trial. Lancet.

[51] Koren M.J., Sabatine M.S., Giugliano R.P., Langslet G., Wiviott S.D., Kassahun H., Ruzza A., Ma Y., Somaratne R., Raal F.J. (2017). Long-term Low-Density Lipoprotein Cholesterol–Lowering Efficacy, Persistence, and Safety of Evolocumab in Treatment of Hypercholesterolemia: Results Up to 4 Years From the Open-Label OSLER-1 Extension Study. JAMA Cardiol..

[52] Raal F.J., Hovingh G.K., Blom D., Santos R.D., Harada-Shiba M., Bruckert E., Couture P., Soran H., Watts G.F., Kurtz C. (2017). Long-term treatment with evolocumab added to conventional drug therapy, with or without apheresis, in patients with homozygous familial hypercholesterolaemia: An interim subset analysis of the open-label TAUSSIG study. Lancet Diabetes Endocrinol..

[53] Kastelein J.J.P., Ginsberg H.N., Langslet G., Hovingh G.K., Ceska R., Dufour R., Blom D., Civeira F., Krempf M., Lorenzato C. (2015). ODYSSEY FH I and FH II: 78 week results with alirocumab treatment in 735 patients with heterozygous familial hypercholesterolaemia. Eur. Heart J..

[54] Blom D.J., Harada-Shiba M., Rubba P., Gaudet D., Kastelein J.J., Charng M.-J., Pordy R., Donahue S., Ali S., Dong Y. (2020). Efficacy and Safety of Alirocumab in Adults with Homozygous Familial Hypercholesterolemia. J. Am. Coll. Cardiol..

[55] Ray K.K., Bruckert E., Peronne-Filardi P., Ebenbichler C., Vogt A., Bridges I., Sibartie M., Dhalwani N. (2023). Long-term persistence with evolocumab treatment and sustained reductions in LDL-cholesterol levels over 30 months: Final results from the European observational HEYMANS study. Atherosclerosis.

[56] Guedeney P., Giustino G., Sorrentino S., E Claessen B., Camaj A., Kalkman D.N., Vogel B., Sartori S., De Rosa S., Baber U. (2022). Efficacy and safety of alirocumab and evolocumab: A systematic review and meta-analysis of randomized controlled trials. Eur. Heart J..

[57] Sabatine M.S., Giugliano R.P., Keech A.C., Honarpour N., Wiviott S.D., Murphy S.A., Kuder J.F., Wang H., Liu T., Wasserman S.M. (2017). Evolocumab and Clinical Outcomes in Patients with Cardiovascular Disease. N. Engl. J. Med..

[58] Schwartz G.G., Steg P.G., Szarek M., Bhatt D.L., Bittner V.A., Diaz R., Edelberg J.M., Goodman S.G., Hanotin C., Harrington R.A. (2018). Alirocumab and Cardiovascular Outcomes after Acute Coronary Syndrome. N. Engl. J. Med..

[59] Tsimikas S. (2018). RNA-targeted therapeutics for lipid disorders. Curr. Opin. Lipidol..

[60] Watts G.F., Raal F.J., Chan D.C. (2022). Transcriptomic therapy for dyslipidemias utilizing nucleic acids targeted at ANGPTL3. Futur. Cardiol..

[61] Hofherr A., Schumi J., Rudvik A., Vega R., Ryden-Bergsten T., Hurt-Camejo E., Johanson P., Carlsson B.C. (2022). Abstract 11482: The PCSK9-Targeted Antisense Oligonucleotide AZD8233 Reduces LDL-C, ApoB, and Lp(a) in Patients with Dyslipidemia on Statin Treatment—Data from the Phase 2b ETESIAN Study. Circulation.

[62] Early Results Show Promise of an Oral PCSK9 Inhibitor. https://www.healio.com/news/cardiology/20211115/early-results-show-promise-of-an-oral-pcsk9-inhibitor.

[63] Abbasi J. (2022). Highlights From the American Heart Association’s Scientific Sessions—ApoB as a Risk Marker, an Oral PCSK9 Inhibitor, and Aspirin and Dementia. JAMA.

[64] Ballantyne C.M., Banka P., Mendez G., Garcia R., Rosenstock J., Rodgers A., Mendizabal G., Mitchel Y., Catapano A.L. (2023). Phase 2b Randomized Trial of the Oral PCSK9 Inhibitor MK-0616. J. Am. Coll. Cardiol..

[65] Gennemark P., Walter K., Clemmensen N., Rekić D., Nilsson C.A., Knöchel J., Hölttä M., Wernevik L., Rosengren B., Kakol-Palm D. (2021). An oral antisense oligonucleotide for PCSK9 inhibition. Sci. Transl. Med..

[66] Kersten S. (2017). Angiopoietin-like 3 in lipoprotein metabolism. Nat. Rev. Endocrinol..

[67] Conklin D., Gilbertson D., Taft D.W., Maurer M.F., Whitmore T.E., Smith D.L., Walker K.M., Chen L.H., Wattler S., Nehls M. (1999). Identification of a Mammalian Angiopoietin-Related Protein Expressed Specifically in Liver. Genomics.

[68] Chen P.Y., Gao W.Y., Liou J.W., Lin C.Y., Wu M.J., Yen J.H. (2021). Angiopoietin-Like Protein 3 (Angptl3) Modulates Lipoprotein Metabolism and Dyslipidemia. Int. J. Mol. Sci..

[69] Harada M., Yamakawa T., Kashiwagi R., Ohira A., Sugiyama M., Sugiura Y., Kondo Y., Terauchi Y. (2021). Association between ANGPTL3, 4, and 8 and lipid and glucose metabolism markers in patients with diabetes. PLoS ONE.

[70] Kersten S. (2021). ANGPTL3 as therapeutic target. Curr. Opin. Infect. Dis..

[71] Raal F.J., Rosenson R.S., Reeskamp L.F., Hovingh G.K., Kastelein J.J.P., Rubba P., Ali S., Banerjee P., Chan K.-C., Gipe D.A. (2020). Evinacumab for Homozygous Familial Hypercholesterolemia. N. Engl. J. Med..

[72] Gaudet D., Gipe D.A., Pordy R., Ahmad Z., Cuchel M., Shah P.K., Chyu K.-Y., Sasiela W.J., Chan K.-C., Brisson D. (2017). ANGPTL3 Inhibition in Homozygous Familial Hypercholesterolemia. N. Engl. J. Med..

[73] Banerjee P., Chan K.-C., Tarabocchia M., Benito-Vicente A., Alves A.C., Uribe K.B., Bourbon M., Skiba P.J., Pordy R., Gipe D.A. (2019). Functional Analysis of LDLR (Low-Density Lipoprotein Receptor) Variants in Patient Lymphocytes to Assess the Effect of Evinacumab in Homozygous Familial Hypercholesterolemia Patients with a Spectrum of LDLR Activity. Arter. Thromb. Vasc. Biol..

[74] Raal F.J., Rosenson R.S., Reeskamp L.F., Hovingh G.K., Kastelein J.J., Rubba P., Ali S., Banerjee P., Chan K.-C., Khilla N. (2020). Abstract 14407: The Longer-term Efficacy and Safety of Evinacumab in Patients with Homozygous Familial Hypercholesterolemia. Circulation.

[75] Ahmad Z., Pordy R., Rader D.J., Gaudet D., Ali S., Gonzaga-Jauregui C., Ponda M.P., Shumel B., Banerjee P., Dunbar R.L. (2021). Inhibition of Angiopoietin-Like Protein 3 with Evinacumab in Subjects with High and Severe Hypertriglyceridemia. J. Am. Coll. Cardiol..

[76] Ward N.C., Chan D.C., Watts G.F. (2022). A Tale of Two New Targets for Hypertriglyceridaemia: Which Choice of Therapy?. Biodrugs.

[77] Watts G., Schwabe C., Scott R., Gladding P., Sullivan D., Baker J., Clifton P., Given B., Hamilton J., Melquist S. (2022). RNA interference targeting hepatic angiopoietin-like protein 3 results in prolonged reductions in serum triglyceride and non-HDL-cholesterol concentrations: First human results with ARO-ANG3. Res. Sq..

[78] Rosenson R.S. ARO-ANG3, an Investigational RNAi Therapeutic, Decreases Serum Angiopoietin-like Protein 3, Triglycerides, and Cholesterol in Patients with Mixed Dyslipidemia 2022. https://ir.arrowheadpharma.com/static-files/5594ffac-512b-42f7-bd6f-8e0effe74933.

[79] Raal F., Bergeron J., Watts G.F., Gaudet D., Sullivan D., Turner T., Hegeleg R.A., Ballantyneh C.M., Knowlesi J.W., Goldberg I. ARO-ANG3, an Investigational RNAi Therapeutic, Decreases Serum LDL-Cholesterol, Apolipoprotein B, and Angiopoietin-like Protein 3 in Patients with Homozygous Familial Hypercholesterolaemia. EAS Abstract 1495. https://ir.arrowheadpharma.com/static-files/93c32012-148d-4cb6-9a2c-e42caf345805.

[80] Gaudet D., Karwatowska-Prokopczuk E., Baum S.J., Hurh E., Kingsbury J., Bartlett V.J., Figueroa A.L., Piscitelli P., Singleton W., Witztum J.L. (2020). Vupanorsen, an N-acetyl galactosamine-conjugated antisense drug to *ANGPTL3* mRNA, lowers triglycerides and atherogenic lipoproteins in patients with diabetes, hepatic steatosis, and hypertriglyceridaemia. Eur. Heart J..

[81] Blom D.J., Raal F.J., Santos R.D., Marais A.D. (2019). Lomitapide and Mipomersen—Inhibiting Microsomal Triglyceride Transfer Protein (MTP) and apoB100 Synthesis. Curr. Atheroscler. Rep..

[82] Musunuru K. (2022). Moving toward genome-editing therapies for cardiovascular diseases. J. Clin. Investig..

[83] Jinek M., Chylinski K., Fonfara I., Hauer M., Doudna J.A., Charpentier E. (2012). A Programmable dual-RNA-guided DNA endonuclease in adaptive bacterial immunity. Science.

[84] Katzmann J.L., Cupido A.J., Laufs U. (2022). Gene Therapy Targeting *PCSK9*. Metabolites.

[85] Cohen J.C., Hobbs H.H. (2006). Sequence Variations in PCSK9, Low LDL, and Protection against Coronary Heart Disease. N. Engl. J. Med..

[86] Musunuru K., Chadwick A.C., Mizoguchi T., Garcia S.P., DeNizio J.E., Reiss C.W., Wang K., Iyer S., Dutta C., Clendaniel V. (2021). In vivo CRISPR base editing of PCSK9 durably lowers cholesterol in primates. Nature.

[87] Wang L., Smith J., Breton C., Clark P., Zhang J., Ying L., Che Y., Lape J., Bell P., Calcedo R. (2018). Meganuclease targeting of PCSK9 in macaque liver leads to stable reduction in serum cholesterol. Nat. Biotechnol..

[88] Rothgangl T., Dennis M.K., Lin P.J.C., Oka R., Witzigmann D., Villiger L., Qi W., Hruzova M., Kissling L., Lenggenhager D. (2021). In vivo adenine base editing of PCSK9 in macaques reduces LDL cholesterol levels. Nat. Biotechnol..

[89] Wang L., Breton C., Warzecha C.C., Bell P., Yan H., He Z., White J., Zhu Y., Li M., Buza E.L. (2021). Long-term stable reduction of low-density lipoprotein in nonhuman primates following in vivo genome editing of PCSK9. Mol. Ther..

[90] Musunuru K., Pirruccello J.P., Do R., Peloso G.M., Guiducci C., Sougnez C., Garimella K.V., Fisher S., Abreu J., Barry A.J. (2010). Exome Sequencing, *ANGPTL3* Mutations, and Familial Combined Hypolipidemia. N. Engl. J. Med..

[91] Chadwick A.C., Evitt N.H., Lv W., Musunuru K. (2018). Reduced Blood Lipid Levels with In Vivo CRISPR-Cas9 Base Editing of ANGPTL3. Circulation.

[92] Nettersheim F.S., De Vore L., Winkels H. (2020). Vaccination in Atherosclerosis. Cells.

[93] Sahebkar A., Momtazi-Borojeni A.A., Banach M. (2021). PCSK9 vaccine: So near, yet so far!. Eur. Heart J..

[94] Momtazi-Borojeni A.A., Jaafari M.R., Afshar M., Banach M., Sahebkar A. (2021). PCSK9 immunization using nanoliposomes: Preventive efficacy against hypercholesterolemia and atherosclerosis. Arch. Med. Sci..

[95] Jennings G.T., Bachmann M.F. (2009). Immunodrugs: Therapeutic VLP-Based Vaccines for Chronic Diseases. Annu. Rev. Pharmacol. Toxicol..

[96] Galabova G., Brunner S., Winsauer G., Juno C., Wanko B., Mairhofer A., Lührs P., Schneeberger A., von Bonin A., Mattner F. (2014). Peptide-Based Anti-PCSK9 Vaccines—An Approach for Long-Term LDLc Management. PLoS ONE.

[97] Crossey E., Amar M.J., Sampson M., Peabody J., Schiller J.T., Chackerian B., Remaley A.T. (2015). A cholesterol-lowering VLP vaccine that targets PCSK9. Vaccine.

[98] Fowler A., Sampson M., Remaley A.T., Chackerian B. (2021). A VLP-based vaccine targeting ANGPTL3 lowers plasma triglycerides in mice. Vaccine.

[99] Fukami H., Morinaga J., Nakagami H., Hayashi H., Okadome Y., Matsunaga E., Kadomatsu T., Horiguchi H., Sato M., Sugizaki T. (2021). Vaccine targeting ANGPTL3 ameliorates dyslipidemia and associated diseases in mouse models of obese dyslipidemia and familial hypercholesterolemia. Cell Rep. Med..

